# Difficult laparoscopic cholecystectomy and preoperative predictive factors

**DOI:** 10.1038/s41598-021-81938-6

**Published:** 2021-01-28

**Authors:** Giuseppe Di Buono, Giorgio Romano, Massimo Galia, Giuseppe Amato, Elisa Maienza, Federica Vernuccio, Giulia Bonventre, Leonardo Gulotta, Salvatore Buscemi, Antonino Agrusa

**Affiliations:** 1 Department of Surgical, Oncological and Oral Sciences, University of Palermo, Via L. Giuffrè, 5, 90127 Palermo, Italy; 2 Department of Radiology, University of Palermo, Palermo, Italy

**Keywords:** Biliary tract disease, Digestive signs and symptoms

## Abstract

Laparoscopic cholecystectomy (LC) is the standard technique for treatment of gallbladder disease. In case of acute cholecystitis we can identify preoperative factors associated with an increased risk of conversion and intraoperative complications. The aim of our study was to detect preoperative laboratory and radiological findings predictive of difficult LC with potential advantages for both the surgeons and patients in terms of options for management. We designed a retrospective case–control study to compare preoperative predictive factors of difficult LC in patients treated in emergency setting between January 2015 and December 2019. We included in the difficult LC group the surgeries with operative time > 2 h, need for conversion to open, significant bleeding and/or use of synthetic hemostats, vascular and/or biliary injuries and additional operative procedures. We collected 86 patients with inclusion criteria and difficult LC. In the control group, we selected 86 patients with inclusion criteria, but with no operative signs of difficult LC. The analysis of the collected data showed that there was a statistically significant association between WBC count and fibrinogen level and difficult LC. No association were seen with ALP, ALT and bilirubin values. Regarding radiological findings significant differences were noted among the two groups for irregular or absent wall, pericholecystic fluid, fat hyperdensity, thickening of wall > 4 mm and hydrops. The preoperative identification of difficult laparoscopic cholecystectomy provides an important advantage not only for the surgeon who has to perform the surgery, but also for the organization of the operating block and technical resources. In patients with clinical and laboratory parameters of acute cholecystitis, therefore, it would be advisable to carry out a preoperative abdominal CT scan with evaluation of features that can be easily assessed also by the surgeon.

## Background

Laparoscopic cholecystectomy (LC) is the gold standard technique for treatment of gallbladder disease in both elective and emergency surgery^1^ because it is associated with fewer postoperative complications and shorter hospital stay^2–6^. Tokyo guidelines^7^ divided acute cholecystitis in three different grades of severity and several studies showed that Grade III acute cholecystitis were associated with increased risk of vasculobiliary injuries and a higher conversion rate to open surgery. The authors identified preoperative factors associated with prolonged operative time and conversion rate (gallbladder wall thickening, C-reactive protein levels, body temperature, age, BMI and previous abdominal surgery). In this classification, the radiologic findings were primarily based on ultrasonography and the laboratory tests evaluated C-reactive protein. On this background, the aim of our study was to identify other preoperative laboratory and radiological features predictive of difficult LC. The knowledge of reliable preoperative predicting factors could be an advantage for both the surgeon and patient in terms of options for management (skills of surgeon, need for intraoperative cholangiography, operative timing) in order to avoid intraoperative complications (vasculobiliary injuries, conversion to open surgery) and to obtain better postoperative outcomes^8–12^. We designed a retrospective case–control study to compare preoperative predictive factors of difficult LC in patients treated in an emergency setting.

## Methods

### Patients selection

This study included patients undergone to LC for acute cholecystitis at the Department of General and Emergency Surgery of the University Hospital Policlinico of Palermo between January 2015 and December 2019. The patients were retrospectively selected from the electronic medical records and all procedures were performed by the same surgical team experienced in LC (more than 500 LC and more than 100 other laparoscopic procedures) in order to compare standard surgical performances^13–15^. Inclusion criteria were the early LC (within 48 h from hospital admission) in an emergency setting for acute cholecystitis and the use of preoperative CT contrast-enhanced abdominal scan. As exclusion criteria we considered the discharge diagnosis for other clinical conditions different from acute cholecystitis, absence of preoperative CT scan, LC performed during other surgical procedures and previous upper abdominal open surgery. We selected 223 patients with inclusion criteria and we designed an observational case–control study with the enrolled patients divided into two groups that were homogeneous for demographic data, but different for surgical reports. We comprised in the difficult LC group (case group) the surgeries with operative time > 2 h, need for conversion to open surgery, significant bleeding and/or use of synthetic hemostats, vascular and/or biliary injuries and additional procedures (intraoperative common bile duct exploration or ERCP). We collected 86 patients with inclusion criteria and intraoperative findings of difficult LC. For control group we used a propensity score matching to check the consistency of the clinical findings using the following variables: age, gender, ASA score, timing for surgery. With a 1:1 ratio we allocated in the control group the last 86 patients obtained with propensity score matching and with preoperative inclusion criteria, but with no operative signs of difficult LC.

### Preoperative data

We registered the preoperative patients data (age, gender), the presence of comorbidities with American Society of Anesthesiology (ASA) score and the laboratory tests (WBC, AST, ALT, alkaline phosphatase, total bilirubin, C-reactive protein and fibrinogen) in order to obtain a complete clinical assessment^16^. In this study we considered only laboratory parameters (WBC, AST, ALT, alkaline phosphatase, total bilirubin, C-reactive protein) congruent with acute cholecystitis and biliary diseases. In addition we used serum fibrinogen because it is part of the coagulation screening of our hospital and was dosed in all patients to undergo surgery. For the opposite reason we did not dose procalcitonin in acute cholecystitis because it is more expensive than C-reactive protein. The features of CT contrast-enhanced abdominal scan were reviewed by two external experienced radiologists^17,18^ blinded respect to laboratory results. We analyzed the presence/absence of gas in the wall and/or lumen of gallbladder, intraluminal membranes, irregular or absent wall, submucosal edema, pericholecystic fluid/pericholecystic abscess, hyperdensity of the fat surrounding the gallbladder, thickening of wall (> 4 mm) and hydrops.

### Surgical technique

LC was performed using the standard French position. We achieved pneumoperitoneum by open technique with a trans-umbilical Hasson’s trocar^19,20^. At the beginning of surgery we respectively positioned others two trocars in left hypochondrium (10 mm) and in right flank (5 mm). In difficult cases we added a fourth trocar in xiphoidal region (5 mm)^15^. The surgical dissection of the Calot’s triangle was conducted using both the direct infundibular approach and the critical view of safety on the basis of local inflammatory findings. Usually, in LC we used monopolar hook for precise dissection, but in an emergency setting we also preferred blunt dissection to prevent vascular or biliary injuries and to take advantage of anatomical planes concealed from inflammatory response. After identification, cystic duct and cystic artery were clipped and divided and we performed retrograde cholecystectomy.

### Statistical analysis

We registered the patients characteristics, preoperative laboratory parameters (WBC, AST, ALT, alkaline phosphatase, total bilirubin, C-reactive protein and fibrinogen) and CT abdominal scan findings (gas in the wall or lumen of gallbladder, intraluminal membranes, irregular or absent wall, pericholecystic fluid/abscess, submucosal edema, hyperdensity of the fat surrounding the gallbladder, thickening of wall and hydrops). We compared these preoperative data between the two groups. Statistical analysis was performed with SPSS 25.0 (SPSS Inc., Chicago, USA). Quantitative variables were expressed as mean ± standard deviation and categorical variables as count (percentage). Continuous data were analyzed with Student t test and categorical variables were compared with Chi-square test. A *p* value < 0.05 was considered statistically significant.

### Ethics approval

Consent to publish was given by University of Palermo.

### Informed consent

All methods were carried out in accordance with relevant guidelines and regulations. All experimental protocols were approved by University of Palermo. Written informed consent was obtained from all patients involved in the study.

## Results

Between January 2015 and December 2019 we selected 223 patients undergone to LC with inclusion criteria. In the difficult LC group (cases) we had 86 patients, 45 male and 41 female, male/female ratio 1:1.1, mean age 65.51 ± 13.49. In the control group we enrolled 86 patients, 39 male and 47 female, male/female ratio 1:1.2, mean age 55.47 ± 16.16. From the analysis of our laboratory data we noted that only WBC, AST and fibrinogen differences between the two groups were statistically significant. The results of each group are shown in Table 1. WBC was 11.06 × 10^3^/µL (SD: 4.55) in the case group and 8.35 × 10^3^/µL (SD: 3.35) in the control group. We demonstrated that a higher WBC count was associated with difficult LC with statistical significance (*p* value < 0.0001); fibrinogen was respectively 466.95 U/L (SD: 210.19) in the case group and 368.84 U/L (SD: 148.55) in the control group (*p* value 0.006). In our analysis total bilirubin was 1.81 mg/dL (SD:1.84) in the cases group and 1.29 mg/dL (SD: 0.99) in the control group and there were no significant differences, such as for levels of ALT (case group: 76.28 U/L SD: 86.36; control group:103.5 U/L, SD:130.49), ALP (case group: 113.78 U/L, SD: 72.44; control group:110.93 U/L, SD: 69.73) and CRP (case group:113.78 U/L, SD: 72.44; control group: 110.93 U/L, SD: 69.73). A singular result of this study indicated that AST was 45.16 U/L (SD: 51.19) in the cases group and 80.08 U/L (SD: 124.79) in the control group. The difference between the two groups was statistically significant (*p* value: 0.03), so, in our analysis, a lower AST level was associated with a difficult cholecystectomy. CT abdominal scan findings are shown in Table 2. We demonstrated no differences regarding gas in the wall and/or lumen of gallbladder, intraluminal membranes (1.16% in cases and absent in control group) and submucosal edema (4.65% in cases; 2.32% in control group). On the contrary there were significant differences between the two groups for irregular or absent wall (12.79% in cases; 2.33% in control group), pericholecystic fluid (20.93% in cases; 12.79% in control group), fat hyperdensity (39.53% in cases; 12.79% in control group), thickening of wall > 4 mm (58.14% in cases; 27.91% in control group) and hydrops (37.21% in cases and 19.77% in control group).

**Table 1:** Laboratory preoperative parameters between patients with difficult LC and not difficult LC.

	Difficult LC (n. 86) (Mean ± SD)	Not difficult LC (n. 86) (Mean ± SD)	Total (n. 172) (Mean ± SD)	*p* value
***Age***	65.5 ± 13.5	55.5 ± 16.2	60.5 ± 15.7	< 0.001
***Gender***				
*M*	45 (52.3%)	39 (45.3%)	84 (48.8%)	0.719
*F*	41 (47.7%)	47 (54.7%)	88 (51.2%)	
***ASA score***				
*ASA 2*	35 (40.7%)	39 (45.3%)	74 (43%)	0.067
*ASA 3*	51 (59.3%)	47 (54.7%)	98 (57%)	
***WBC***	11.06 ± 4.55	8.35 ± 3.35	9.73 ± 4.12	< 0.001
***Bilirubin***	1.81 ± 1.84	1.29 ± 0.99	1.56 ± 1.52	0.058
***AST***	45.16 ± 51.19	80.08 ± 124.79	62.06 ± 95.5	0.039
***ALT***	76.28 ± 86.36	103.5 ± 130.49	89.34 ± 110.2	0.163
***ALP***	113.78 ± 72.44	110.93 ± 69.73	112.45 ± 70.9	0.825
***CRP***	49.19 ± 79.10	8.7 ± 13.58	38.4 ± 70.9	0.097
***Fibrinogen***	466.95 ± 210.19	368.84 ± 148.55	422.76 ± 190.6	0.006

**Table 2:** Comparison of CT scan findings between difficult LC and not difficult LC groups.

CT scan findings	Difficult LC (n. 86) (%)	Not difficult LC (n. 86) (%)	Total (n. 172) (%)	*p* value
Intraluminal membranes	1 (1%)	0 (0%)	1 (0.6%)	0.38
Irregular or absent wall	11 (13%)	2 (2%)	13 (7.5%)	< 0.0001
Submucosal edema	4 (5%)	2 (2%)	6 (3.5%)	0.24
Pericholecystic fluid	18 (21%)	11 (13%)	29 (16.9%)	0.0003
Fat iperdensity	34 (40%)	11 (13%)	45 (26.2%)	< 0.0001
Thickening of the wall > 4 mm	50 (58%)	24 (28%)	74 (43%)	< 0.0001
Hydrops	32 (37%)	17 (20%)	49 (28.5%)	< 0.0001

## Discussion

The research of predictive preoperative factors for difficult LC is essential to estimate the probability of conversion, to identify high-risk procedures, to optimize the surgical plan and efficiency of the operating room and to change, when needed, the surgical technique or the surgeon. Moreover, the use of predictive factors can allow us to select patients eligible for non-surgical treatment^8,12^. In this study, we retrospectively analyzed preoperative laboratory tests and radiological findings in the two groups (difficult LC and not-difficult LC) in order to evaluate statistically significant differences. The choise of these parameters was related to surgical procedure that in LC comprised two phases: the dissection of the Calot’s triangle with identification of cystic duct and cystic artery and the detachment of gallbladder from the liver. We assumed that the difficulty of LC and the risk of biliovascular injuries derived from the degree of inflammation of the infundibular region during the Calot’s triangle dissection. On these basis, laboratory tests reflected systemic (WBC count, C-reactive protein, fibrinogen) and local (AST, ALT, ALP, bilirubin) inflammatory response and CT scan allowed to obtain precise information about local conditions. We did not consider preoperative clinical predictive factors like number of attacks of pain or prior conservative treatment because the aim of this case–control study is to identify the “real” difficult laparoscopic cholecystectomy with analysis of objective data on surgical reports and to compare objective radiological preoperative features of these patients. We know the comorbidities, number of attacks of pain such as age and/or sex are predictive factors of difficult laparoscopic cholecystectomy but in this study the evaluation of these parameters adds nothing to our analysis^21–23^. From the review of literature we directed our research towards certain risk factors: leukocytosis^9,12^, serum bilirubin, AST, ALT, ALP^8,12^, fibrinogen^8^, C-reactive protein^13^, wall thickening, presence of pericholecystic fluid/abscess, gas in the wall or lumen, intraluminal membranes, irregular or absent gallbladder wall and pericholecystic inflammation^10–12,24–27^. The analysis of the collected data showed that there was a significant association between elevated WBC count and fibrinogen level and difficult LC. This result could be explained with the fact that these laboratory parameters are excellent indices of inflammatory response, and that pericholecystic inflammation was closely related to the difficulty of surgical procedure^28^. Especially with regard to the WBC count, our data are in agreement with a prospective study by Nidoni et al.^9^ that identified a WBC count > 11.000/mm^3^ as a predictive factor of difficult LC. At the same time difficult LC was significant associated with higher fibrinogen levels. In our study there were no statistically significant association among ALP, ALT and bilirubin values and difficult LC, as instead described by Bourgouin et al^8,12^. In this study we found no significant differences regarding C-reactive protein level in discordance with several authors^12,29–31^ that used preoperative C-reactive protein as a predictive factor of difficult LC. The results obtained in our study could be interpreted by the fact that in our analysis all patients had acute cholecystitis with a raise in inflammation indexes. Therefore the increase in C-reactive protein could be less specific compared to other local parameters. For this reason we associated the analysis of CT enhanced abdominal scan. In literature we found only fews articles on the role of CT scan in difficult LC^24,29^. We registered significant differences between the two groups for irregular or absent wall, pericholecystic fluid, surrounding fat iperdensity, thickening of the wall and hydrops. Instead there were no differences about gas in the wall or in the lumen of gallbladder, intraluminal membranes and submucosal edema. The absence of gas in the wall or lumen of gallbladder and the lower number of intraluminal membranes could be due to early surgical treatment of the patients included in the study. On the contrary, the study by Maehira et al.^24^ showed no significant association between CT scan findings and difficult LC, but these results could be related to smaller sample taken into consideration. In this study we considered only eligible patients underwent to early LC (within 48 h from admission) in order to reduce the variables related to duration from the onset of cholecystitis. We did not considered the retrospective design a disadvantage because the analyzed parameters were objectives and registered from medical records. In our opinion, in emergency setting, a prospective study could lead to biased results in terms of conversion rate and vasculobiliary injuries because could unconsciously push those who participate in it to change their management policies. At the same time this observational study can lead to a limitation in terms of patient allocation with perhaps dissimilar groups, but among inclusion criteria the main one was intraoperative "difficulty" of the surgical treatment therefore we tried to minimize this error by considering only patients treated within 48 h from admission and managed by the same surgical team in order to reduce the variables depending on the local inflammatory conditions and on the surgeon experience. This study had other several limitations. This was a single centre study^32–34^ and the retrospective enrollment had potential bias about limited number of selected patients.

## Conclusion

The analysis of literature and the results of this study showed that laboratory parameters and preoperative abdominal CT scan features could provide relevant indications on factors predicting the difficulty of surgery. The preoperative identification of cases of difficult laparoscopic cholecystectomy is an important advantage not only for the surgeon who has to perform the surgery, but also for the organization of the operating room and technical resources. In patients with clinical and laboratory indexes of acute cholecystitis, therefore, it would be advisable to carry out a preoperative study with CT abdominal scan and evaluation of parameters that can be easily assessed also by the surgeon.

## Data Availability

The datasets used and/or analysed during the current study are available from the corresponding author on reasonable request. The datasets used and/or analysed during the current study are available from the Department of Surgical, Oncological and Oral Sciences, University of Palermo, Via L. Giuffrè 5—90,127 Palermo, on reasonable request.

## Abbreviations

LC: Laparosocpic cholecystectomy
WBC: White blood cells
AST: Aspartate aminotransferase
ALT: Alanine aminotransferase
ALP: Alkaline phosphatase
CRP: C-reactive protein
